# Monolithic nano-porous polymer in microfluidic channels for lab-chip liquid chromatography

**DOI:** 10.1186/s40580-018-0151-4

**Published:** 2018-07-10

**Authors:** Jin-young Kim, Danny O’Hare

**Affiliations:** 1 DGIST-ETH Microrobotics Research Center, DGIST, Daegu, 42988 South Korea; 2 Department of Bioengineering, Imperial College London, London, SW7 2AZ UK

## Abstract

In this paper, a nano-porous polymer has been integrated into the microfluidics device as on-chip monolithic liquid chromatography column for separation of chemical and biological samples. Monolithic nano-porous polymer (MNP) was formed and firmly grafted on the surface of the microfluidic channel. Neurotransmitters, 5-hydroxyindole-3-acetic acid (5-HIAA) and 5-hydroxytryptamine (serotonin, 5-HT), were successfully separated with the developed on-chip MNP column.

## Introduction

In recent years, microfabricated systems have been considered as promising tools for chemical and biological analysis because of the rapid analysis and control of minute samples [1–8]. Since separation of chemical components is an important technique in bioanalysis, various trials have been conducted to integrate separation functionality onto the micro-total analysis system (μ-TAS) [9–11]. Liquid chromatography (LC) is the most popular separation technique [12, 13]. In addition, monolithic stationary phases potentially offer the advantages of the simple control of permeability and surface areas and easy preparation for application within a micro-fluidic channel for lab-on-a chip (LOC) [14–17]. In this paper, LC separation columns were formed in TPE channels using nano-porous polymer as a monolithic column. The packed channel was investigated by SEM. In addition, permeability was calculated from back-pressures measured as a function of flow-rates. Neurotransmitters were separated by the developed on-chip monolithic LC column to validate separation performance.

## Methods

### Materials

For microfluidic devices, TPE (CFS Fibreglass, UK) was used due to high mechanical robustness withstanding up to approximately 18 MPa [18], which is suitable for high pressure liquid chromatography. It was prepared by mixing with polymerization catalyst, methyl ethyl ketone peroxide (MEKP). Polyethylene terephthalate (PET) (Daedong Polymer, South Korea) was used as the substrate to seal the TPE microfluidic channel. Next, the mixture of ethylene diacrylate (EDA, monomer) 0.485 g, methyl methacrylate (MMA, cross-linker) 0.485 g and benzophenone (BP, photo-initiator) 0.03 g was prepared for the grafting layer. Butyl methacrylate (BuMA, monomer) 0.6 g, ethylene dimethacrylate (EDMA, cross-linker) 0.4 g, 1-dodecanol (porogen) 1.5 g and 2,2-dimethoxy-2-phenylacetophenone (DMPAP, photo-initiator) 0.01 g were mixed for the monolithic nano-porous polymer. Two neurotransmitters, 5-HIAA and 5-HT, were purchased from Sigma-Aldrich.

### Fabrication procedure

**Fig. 1:**
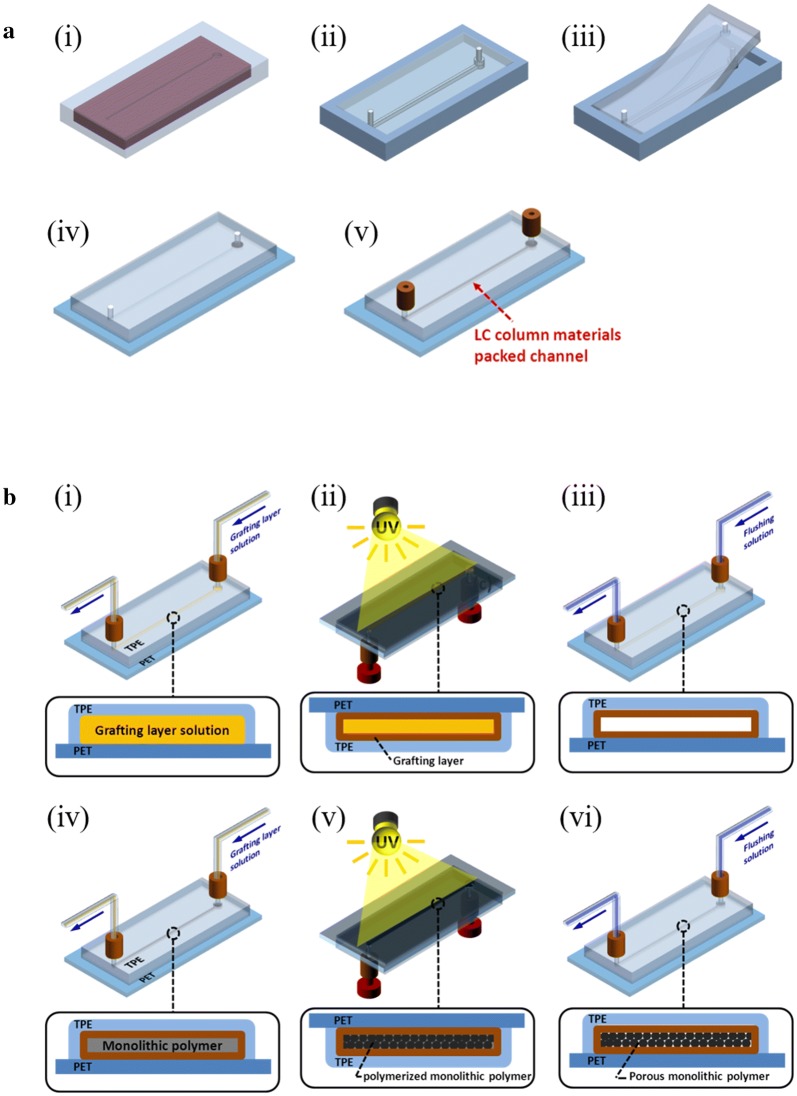
Fabrication procedures of **a** TPE microfluidic device, (i) PDMS replica mould from the acrylate master mould, (ii) TPE pouring, (iii) peeling off the semi-cured TPE, (iv) bonding with the PET substrate, (v) PEEK unions attachment and the channel packing with MNP, and **b** poly(methyl acrylate) monolithic column packing in the channel, (i) introducing a photografting solution into a TPE channel, (ii) UV exposure through PET substrate, (iii) flushing the residual solution by flowing a methanol-based cleaning solution, (iv) introducing the poly(methyl acrylate) mixture solution, (v) UV exposure through PET substrate and (vi) flushing unreacted solutions using the cleaning solution

As shown in Fig. 1a, TPE microfluidic device was fabricated by rapid-prototyping process. After the acrylate master mould for LC separation channel were prepared, the poly(dimethylsiloxane) (PDMS) replica was moulded. PDMS was prepared by mixing a resin and its catalyst with a ratio 10:1 then the mixture was degassed in a vacuum desiccator to remove bubbles. The master mould was placed in square petri-dishes and the prepared PDMS was poured into the dishes 3–4 mm higher than the surface of the master moulds. After the PDMS was levelled, degassing was repeated to ensure an even and bubble free surface for the channels. Next it was cured at 40 °C overnight, since the acrylate mould begins to crack above 50 °C. The fully cured PDMS replica was used as a working mould to cast TPE microfluidic devices. After the PDMS replica mould was fabricated, the TPE microchannel was cast for the robust microfluidic devices. Once the TPE was completely cured, it is difficult to bond to substrates even if the surface treatment is conducted. Therefore, TPE was cured in the two steps. TPE resin and the MEKP catalyst were mixed in a ratio of 100:1 (w/w), degassed and decanted onto the PDMS mould. The resulting structure was partially cured in an oven for 10 min at 60 °C. In the meantime, the PET substrate was sonicated in isopropyl alcohol (IPA) and dried in a stream of N_2_ gas. The PET surface was treated with an O_2_ plasma to obtain strong sealing of TPE microchannels. The semi-cured TPE microchannel that has a jelly-like consistency was separated from the PDMS mould then attached to the PET substrate to seal the microchannels. The semi-cured TPE could easily be removed from PDMS replica mould because of the flexibility of PDMS. Finally, the entire device was heated at 76 °C for 1 h to complete the TPE cure and then cooled down to room temperature over several minutes. Figure 1b describes the schematic of poly(methyl acrylate) monolithic column packing in the TPE channel. Firstly, the TPE channel was blown by N_2_ gas to remove dust or microparticles inside. N_2_ gas was purged for 10 min into the grafting solution to remove oxygen otherwise it can expand and form voids by heat energy during polymerisation. Then the grafting layer solution was introduced into the channel. The inlet and outlet were then gently cap-screwed to keep out air bubbles that can generate voids during polymerisation and significantly reduce the separation efficiency. The TPE device was inverted and UV light [broadband (290–385 nm), 12.22 mW/cm^2^] was radiated to the grafting solution through the PET substrate because TPE over 1 mm thickness absorbs most of UV light [18]. After polymerisation of the thin grafting layer on the channel surface, the channel was flushed by the cleaning solvent [1:1 (v/v) methanol:DI water] with 10 times volume (500 μL) of the channel to remove the unreacted polymer. The grafting layer was blown by N_2_ and dried in 40 °C oven for 1 h. Next, the N_2_ purged monolithic column solution filled the TPE channel and was similarly exposed to UV for 10 min for polymerisation. The remaining porogenic solvent and photo-initiators were flushed out by the cleaning solvent at 10 μL/min for 1 h. The monolithic column in the TPE channel was dried at 40 °C overnight.

### Characterization

The static contact angle was measured using deionized (DI) water to investigate effects of polymerisation condition on the grafting layer properties and optimise the formation of the grafting layer on polymer substrates. The grafting layer was polymerised for different UV exposure times on PET substrate then the surface condition was analysed by contact angle measurement. The grafting solution was spin-coated with 2000 rpm for 30 s on to 2 cm × 2 cm square PET substrates and exposed to UV light. The UV light was the broad band that contains g-line (436 nm), h-line (405 nm) and i-line (365 nm). The grafting layer was illuminated through the PET substrate to mimic the grafting conditions found within the microchannel, where UV light is always irradiated to the solution through the bottom substrate, PET due to the low UV transmittance of TPE. UV exposure time was varied from 0 to 30 min. Then samples were dried overnight in an oven at 40 °C. The static contact angle was measured with deionised water.

The high back pressure results in problems such as the rupture of columns or chips. Therefore, it should be as low as possible. The back pressure can be interpreted as the permeability of the column for the mobile phase. It is related to the amount of the macropores and the surface area on the stationary phase. While a large number of micropores (< 2 nm) and mesopores (2–50 nm) should be introduced into the polymer in order to create a large surface area [14], large macropores with diameters over 50 nm make a contribution to the permeability rather than the overall surface area. In simple terms, a balance between low flow resistance and large surface area is necessary. The pore size distributions can be carefully controlled by the optimization of the polymerisation conditions [14]. Permeability of the separation column can be calculated from back-pressures as a function of flow-rates or linear velocity in a column. DI water was used as a mobile phase and pumped into the column at the flow-rates from 0 to 500 μL/min. Back pressure was measured after 5 min stabilisation time, whenever the flow-rate was changed. The recorded back pressure [also called the pressure-drop (∆P)] was converted to permeability (k) by Darcy’s equation,

1 $$ Q = \frac{{ - kA\left( {P_{b} - P_{a} } \right)}}{\mu L} $$

where Q is the total flow rate, A is the cross-sectional area of a column, µ is the dynamic viscosity and L is the length of a column. The negative sign indicates the inlet pressure (P_a_) is higher than the outlet pressure (P_b_).

As a proof of concept, neurotransmitters, 5-HIAA and 5-HT, were separated using the fabricated on-chip MNP column and HPLC system (Agilent HP 1050, USA).

## Results and discussion

**Fig. 2:**
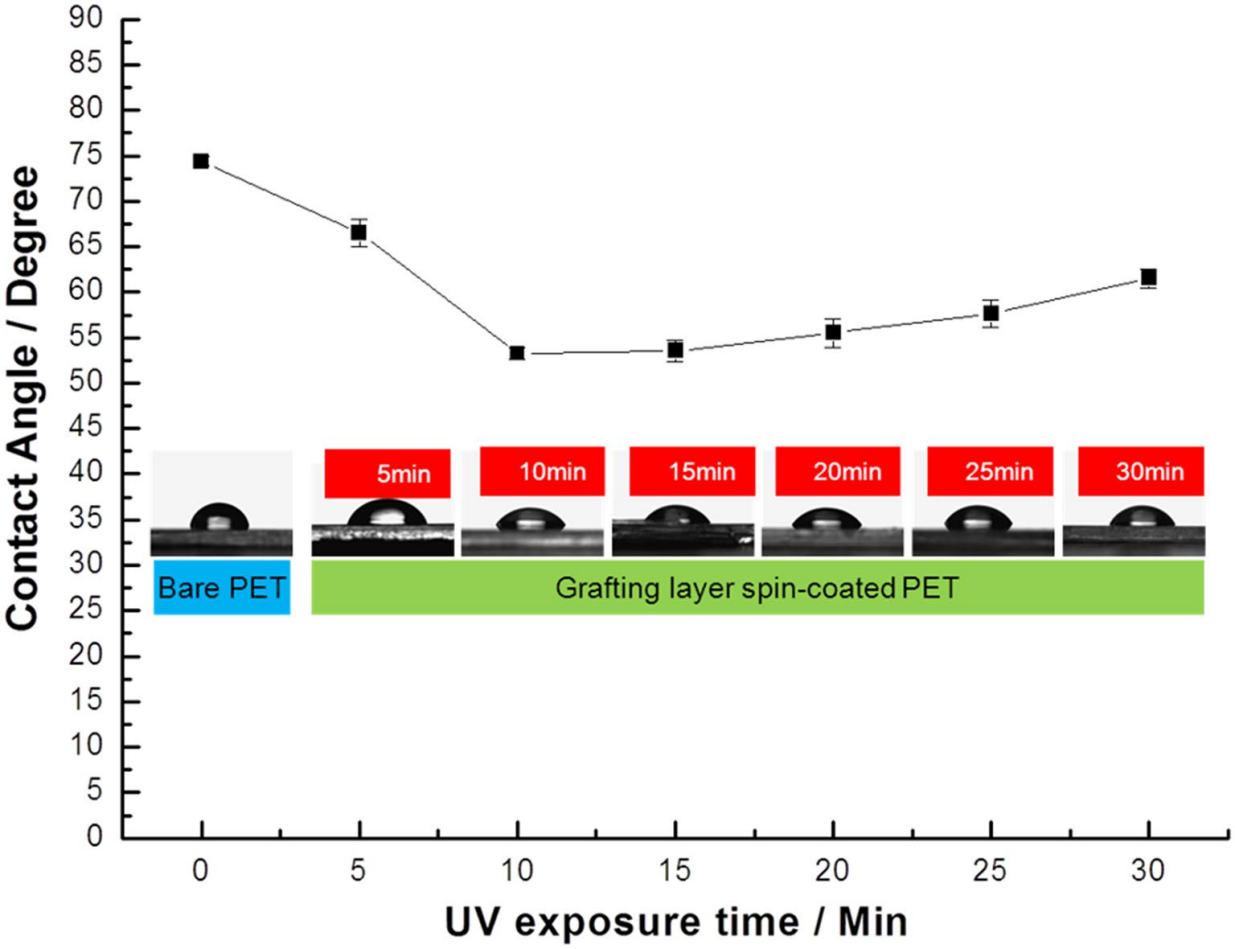
The contact angle measurement of the grafting layer on PET substrate

Overall, a lower contact angle was observed on the PET substrates with the grafting layer in comparison to the bare PET substrate, as shown in Fig. 2. The bare PET substrate has about 75° contact angle, whereas all the grafted PET samples showed approximately from 55° to 65° due to the higher surface energy by the covalent anchors of the grafting layer. In terms of the grafted PET samples, the contact angle began to decrease from 5 min UV exposure and the lowest contact angle was measured on the sample formed by 10 min UV exposure. For the rest of samples, it slightly increased (~ 5°). It may be because that its polymerisation is triggered by UV light on the PET substrate and the reactive chains are created. Then the chains grow during 10 min UV exposure, branching and cross-linking each other. The number of chains approaches the maximum at 10 min then slowly reduces because of probably excessive cross-linking. As a result, 10 min was decided as the UV exposure time for the grafting layer.

**Fig. 3:**
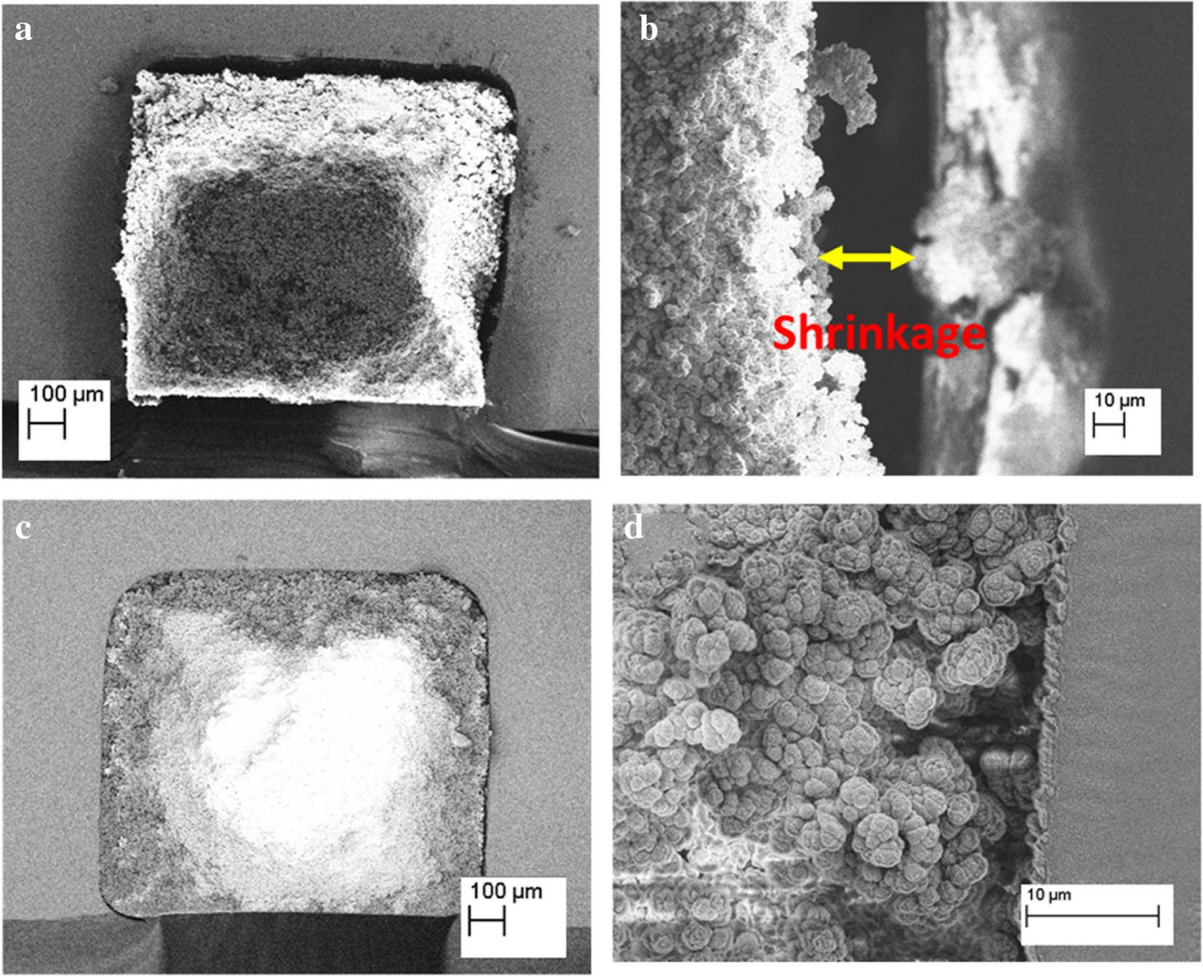
The poly(methyl acrylate) monolith **a** without and **b** with the grafting layer

The grafting layer enables covalent bonding of monoliths on the polymer channel surface. As shown in Fig. 3, significant shrinkage of the monolith was observed within the bare TPE channel, whereas the monolith was firmly bonded with the grafting layer. The importance of the grafting layer was obviously confirmed in this characterisation.

**Fig. 4:**
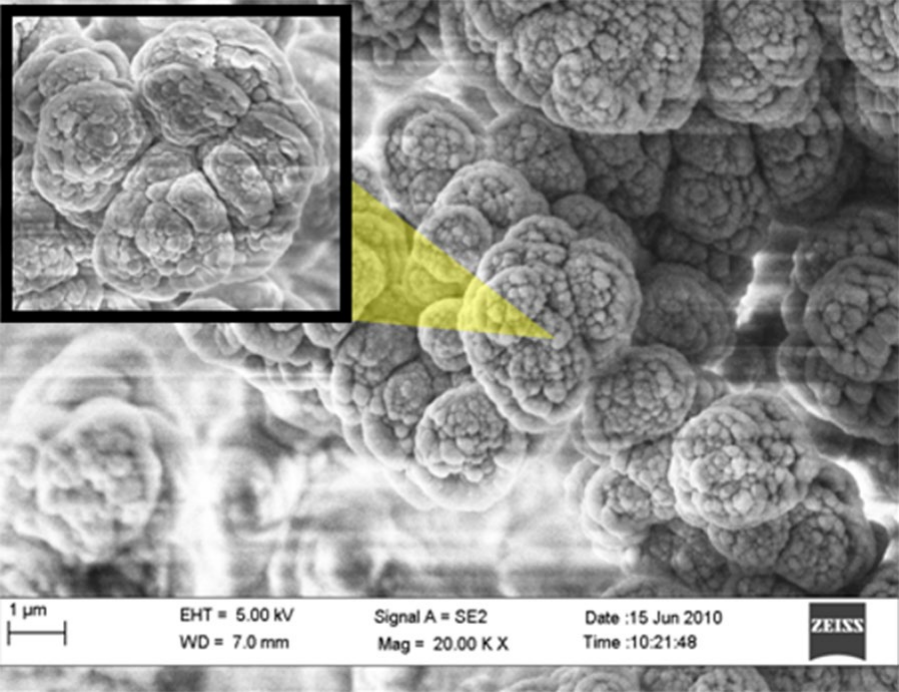
The poly(methyl acrylate) monolith column in the microfludic channel **a**, **b** without and **c**, **d** with the grafting layer. (b) and (d) shows the interface between the monolith and the surface of the microfluidic channel

The monolith consists of a number of globes that are cross-linked to each other and the globular structure includes numerous mesopores between the globes (Fig. 4). Furthermore, micropores were observed on the surface of the globes. These pores significantly contribute to increase the surface areas of the stationary phase. Each globe is approximately 2 µm in diameter and the monolith is bonded well on the channel wall by the grafting layer.

**Fig. 5:**
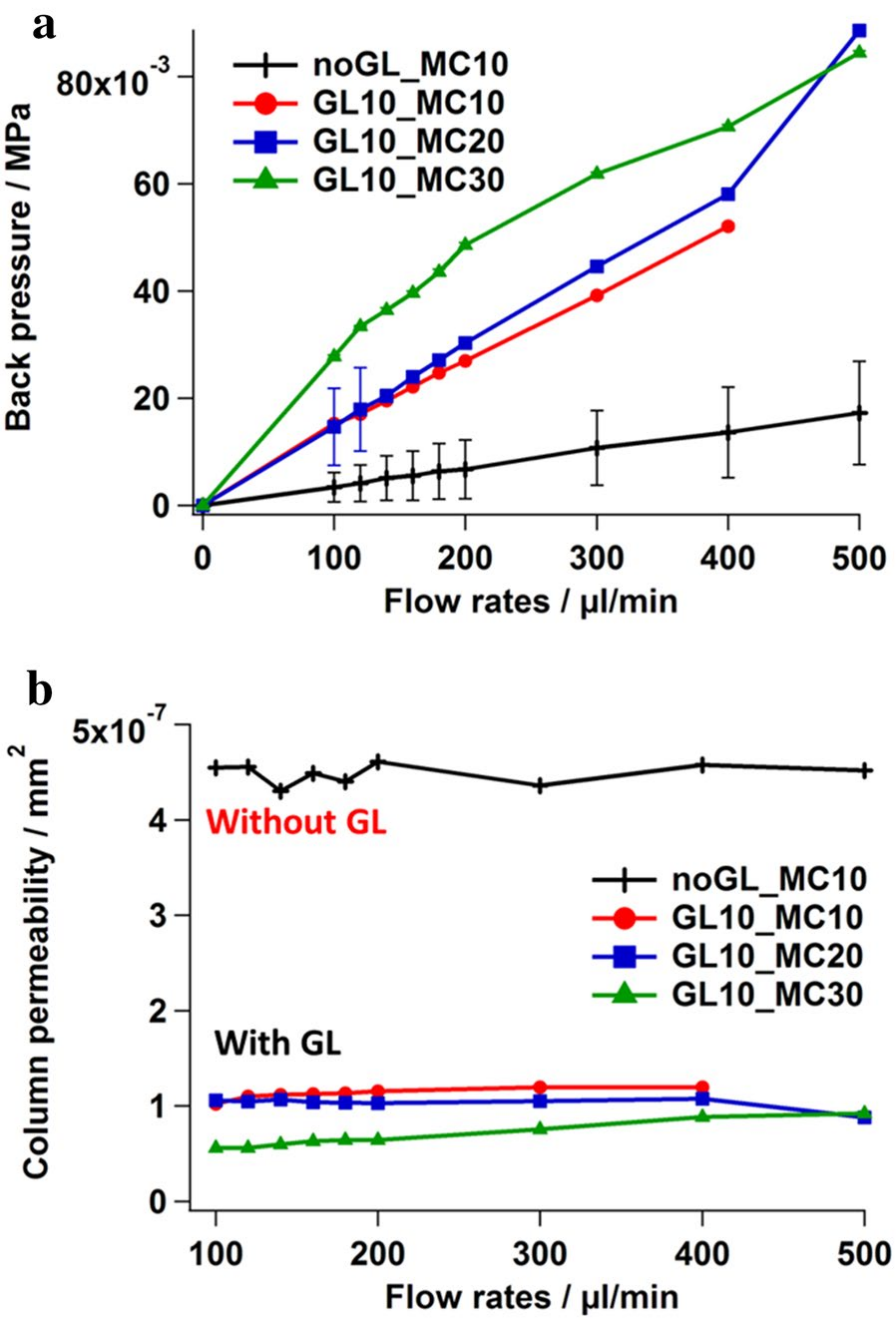
Packing efficiency test of the poly(methyl acrylate) monolithic TPE column without and without the grafting layer, **a** back pressure and **b** column permeability as a function of the flow rate

In case of permeability of the monolithic TPE column, the monolithic TPE column without the grafting layer was compared to that with the grafting layer. Furthermore, the monolith was prepared by 10, 20 and 30 min polymerisation in order to test the effect of the UV exposure time on the column. Figure 5 shows back-pressure and permeability of those columns as a function of the flow rate. The back pressure was proportional to the flow rate in all columns. However, a four times higher pack pressure resulted from the monolithic TPE column (10 min UV polymerisation) with the grafting layer rather than the column without the grafting layer. The grafted monolithic column showed up to 0.05 MPa back pressure at 400 µL/min, whereas the non-grafted column showed approximately 0.017 MPa at the flow-rate.

As shown in Fig. 5b, the permeability of the grafted column was also about 4 times lower, showing approximately 1 × 10^−7^ mm^2^. In contrast, 4.5 × 10^−7^ mm^2^ permeability was obtained from the non-grafted column. This result explains that the monolith packing in the TPE channel was improved by the grafting layer due to the covalent bonding, preventing the shrinkage. UV exposure time did not significantly affect the permeability, showing about 1 × 10^−7^ mm^2^ ± 1.78 × 10^−8^.

**Fig. 6:**
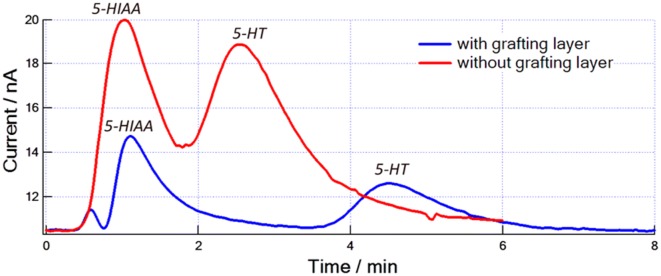
Separation of neurotransmitters mixture at 150 µL/min using the poly(methyl acrylate) monolithic TPE column. [solutes: 5-HIAA and 5-HT(serotonin)]

Separation performance was compared between the non-grafted and grafted monolithic TPE columns using two neurochemicals [5-HIAA and serotonin (5-HT)]. As shown in Fig. 6, those were separated properly with base-line resolution (Rs = 2.16) in the grafted monolithic column, whereas the non-grafted monolithic column showed poor resolution (Rs = 0.55). The performance was improved in overall separation parameters. It is because of the void between the monolith and the channel wall that are shown in Fig. 3. This result confirmed that the grafting layer is necessary for better performance in the monolithic column.

## Conclusions

On-chip MNP column was successfully fabricated by packing channels with poly(methyl acrylate) monolithic nano-porous polymer. The MNP column was firmly bonded on the channel wall without shrinkage by grafting layer, forming 2 μm diameter globes. The grafted MNP column showed better separation performance (Rs = 2.16) than the non-grafted (Rs = 0.55) because of the covalent bonding of MNP onto the microfluidic channel surface without gap.
